# Translatome Regulation in Neuronal Injury and Axon Regrowth

**DOI:** 10.1523/ENEURO.0276-17.2018

**Published:** 2018-05-10

**Authors:** Meir Rozenbaum, Marek Rajman, Ida Rishal, Indrek Koppel, Sandip Koley, Katalin F. Medzihradszky, Juan A. Oses-Prieto, Riki Kawaguchi, Paul S. Amieux, Alma L. Burlingame, Giovanni Coppola, Mike Fainzilber

**Affiliations:** 1 Department of Biomolecular Sciences, Weizmann Institute of Science, Rehovot 76100, Israel; 2 Department of Pharmaceutical Chemistry, University of California San Francisco, San Francisco, CA 94158-2517; 3 Departments of Psychiatry and Neurology, Semel Institute for Neuroscience and Human Behavior, University of California Los Angeles, Los Angeles, CA 90095; 4 Department of Pharmacology, University of Washington, Seattle, WA 98195

**Keywords:** axon growth, axon injury, nerve regeneration, translational regulation

## Abstract

Transcriptional events leading to outgrowth of neuronal axons have been intensively studied, but the role of translational regulation in this process is not well understood. Here, we use translatome analyses by ribosome pull-down and protein synthesis characterization by metabolic isotopic labeling to study nerve injury and axon outgrowth proteomes in rodent dorsal root ganglia (DRGs) and sensory neurons. We identify over 1600 gene products that are primarily translationally regulated in DRG neurons after nerve injury, many of which contain a 5’UTR cytosine-enriched regulator of translation (CERT) motif, implicating the translation initiation factor Eif4e in the injury response. We further identified approximately 200 proteins that undergo robust de novo synthesis in the initial stages of axon growth. ApoE is one of the highly synthesized proteins in neurons, and its receptor binding inhibition or knockout affects axon outgrowth. These findings provide a resource for future analyses of the role of translational regulation in neuronal injury responses and axon extension.

## Significance Statement

Translatome analyses are used to identify a large cohort of genes that are primarily translationally regulated after nerve injury. Many of these contain a 5'UTR cytosine-enriched regulator of translation (CERT) motif, suggesting that the translation initiation factor Eif4e may be involved in the injury response. A number of gene products are robustly synthesized in growing axons. The findings suggest that translational events regulate the neuronal injury response.

## Introduction

Axonal injury to peripheral neurons elicits a sequence of molecular and cellular events that are required for a successful regenerative response (Rishal and Fainzilber, 2014; Terenzio et al., 2017). Information on the lesion is conveyed to the cell body via retrograde calcium waves or macromolecular transport complexes (Perry et al., 2012; Shin et al., 2012; Cho et al., 2013). These retrograde signals elicit extensive transcriptional responses on arrival at the neuronal cell body (Michaelevski et al., 2010b; Ben-Yaakov et al., 2012), reflected by the large number of regeneration-associated genes that have been described in different paradigms of neuronal injury (Dulin et al., 2015; Chandran et al., 2016; Tasdemir-Yilmaz and Segal, 2016). Transcriptional regulation has been the main focus to date in the analysis of injury-induced changes in gene expression, while the role of translation has been examined mainly in the context of localized responses in the axon (Zheng et al., 2001; Michaelevski et al., 2010a; Jung et al., 2012; Twiss et al., 2016). Does initiation of regeneration require the neuronal cell body to change patterns of translation in addition to or differently from the transcriptional regulation that is known to occur?

An early study showed that cultured sensory neurons can extend new neurites in the absence of ongoing transcription during the first phase of outgrowth *in vitro*, but require new transcription for longer term elongating growth (Smith and Skene, 1997). Others described a role for ribosomal protein translation in axon growth under transcriptional repression (Twiss et al., 2000), and more recent studies have implicated translational repression and ribosome biogenesis as rate-limiting mechanisms for axon or dendrite growth (Slomnicki et al., 2016; Williams et al., 2016). A number of studies have shown that axon growth requires local protein synthesis (Zheng et al., 2001; Jiménez et al., 2005; Verma et al., 2005) and that changes in the balance between synthesis in axon versus soma affects axonal growth rates (Perry et al., 2016), but the overall contribution of soma translational regulation in axon outgrowth has not been comprehensively characterized to date.

Translating ribosome affinity purification (TRAP) and metabolic labeling are two types of approaches that can provide comprehensive analyses of translatome and proteome dynamics during the injury response. The first uses ribosome pull-downs followed by RNA-sequencing (RNA-seq) to identify RNAs associated with ribosomes in the cell or tissue of interest (Brar and Weissman, 2015). Studies of this nature in the nervous system typically take advantage of genetic tagging of ribosomes in the cell types of interest with GFP or HA epitope tags, to allow pull-down of the desired ribosome population directly from tissue lysates (Heiman et al., 2008; Sanz et al., 2009). This type of approach has been used for molecular profiling of diverse aspects of neuronal physiology, including activity (Tao et al., 2016), development (Shigeoka et al., 2016), maturation (Zhang et al., 2016), and pathology (Sun et al., 2015). However, ribosomal association alone may not necessarily reflect translational activation, since mRNAs may remain associated with ribosomes under translational arrest (Ingolia et al., 2011), and non-coding RNAs are also a prominent component of ribosome pull-downs (Carlevaro-Fita et al., 2016). Hence, metabolic labeling for direct monitoring of protein synthesis can provide a valuable complementary approach.

Metabolic labeling coupled with mass spectrometry analysis provides direct assessment of translational activity on a proteome-wide scale. In the stable isotopic labeling of amino acids in culture (SILAC) approach, differential proteome labeling is achieved by culturing cells in media containing different isotopes of an essential amino acid (Ong et al., 2002). SILAC was originally developed for dividing cells, and uniform labeling may require three to five cell divisions in culture. Although 70% −80% label incorporation has been reported in rapidly growing embryonic neurons after 3 d of culture (Spellman et al., 2008), non-dividing cells such as neurons are more easily assessed by pulsed or multiplex labeling approaches (Hoedt et al., 2014; Zhang et al., 2014). These and other variants of SILAC have been employed to characterize diverse aspects of neuronal cell physiology, such as synaptic protein turnover (Cohen et al., 2013), synapse development (Ultanir et al., 2014), and signaling endosome dynamics (Debaisieux et al., 2016). Here, we employ TRAP, RNA-seq, and SILAC-based methods to interrogate the translational response to nerve injury in dorsal root ganglia (DRGs) and in sensory neurons.

## Materials and Methods

### Animals, injury models, and neuronal cultures

The study was conducted in accordance with the guidelines of the Institutional Animal Care and Use Committee. Male mice at 8–12 weeks of age were used for ribosome pull-down and RNA-seq experiments. The following mouse strains were used: RiboTag (Sanz et al., 2009), *Adv Cre* (Zhou et al., 2010; da Silva et al., 2011), *Isl1 Cre* (Yang et al., 2006), *Dhh Cre* (Jaegle et al., 2003), *Runx3 Cre* (Levanon et al., 2011), *PGK Cre* (Lallemand et al., 1998), and *ApoE null* (Piedrahita et al., 1992). Wistar rats were used for proteomics analyses and *C57BL/6 YFP16* mice (Feng et al., 2000) for functional validation of selected candidates. Cultures of control and conditionally injured primary sensory neurons from mouse or rat DRG were performed as described previously (Hanz et al., 2003; Perry et al., 2012). For proteomics, adult Wistar rats were subjected to sciatic nerve (SN) crush, and L4-6 DRG were isolated for culturing 3 d later. Control and injury-conditioned ganglia were dissociated with 500-U/ml collagenase (Sigma) and grown in Neurobasal A medium with B27 supplement (Invitrogen) containing L-Lysine:2HCl (13C6, 99%; 15N2, 99%, Cambridge Isotope Laboratories) for 24, 30, and 48 h.

Discrimination between axons and cell bodies of neuronal cultures was achieved by culturing the cells in modified Boyden chambers (Willis and Twiss, 2011). In this system, neurons are plated on filter inserts containing a polyethylene tetraphthalate membrane with 3-µm pores (Millipore) that had been coated with poly-L-lysine (Sigma) and laminin (Invitrogen). Neurons from 15–20 DRGs were plated per insert, and axon-enriched protein extracts were prepared (Zheng et al., 2001; Willis and Twiss, 2011). Briefly, cell bodies and non-neuronal cells were removed from the upper membrane surface, followed by extraction of the axon-enriched lower side of the membrane using a modified RIPA buffer [50 mM Tris-HCl (pH 7.4), 150 mM NaCl, 0.5% NP-40, and 0.1% SDS] with protease inhibitors (Roche). Multiple biological replicates corresponding to five to seven animals each were combined and concentrated using centrifugal filters (Amicon Ultra, 3 kDa; Millipore). Protein concentration was measured with the Micro BCA Protein Assay (Thermo Scientific). Equal protein amounts were separated by 1D SDS-PAGE (10%) and stained with Novex Colloidal Blue (Invitrogen). Each gel lane was cut into 15 parts. Gel pieces were reduced, alkylated and digested with trypsin (https://msf.ucsf.edu/protocols.html), followed by on-line high-pressure liquid chromatography/mass spectrometry (LC/MS) using a NanoACQUITY-LTQ-Orbitrap XL system (Waters and Thermo Scientific, respectively). Each digest mixture was analyzed over 90-min fractionation on a C18 reversed-phase nanocolumn. Masses were measured in the Orbitrap, collision-induced dissociation on the six most abundant multiply charged ions of the MS survey was performed in the linear trap, and fragment masses were also detected there.

Transcription inhibition experiments on neuronal cultures were conducted by culturing adult rat DRG neurons with or without the RNA polymerase II inhibitor 5,6-dichloro-1-β-D-ribofuranosylbenzimidazole riboside (DRB; Sigma) at 80 µM final concentration. After 12 h, the DRB-containing medium was removed and cultures were washed twice with warm PBS. Fresh growth medium (DMEM/F12 + 10% HS + N2 + P/S) was then added and cultures were continued for up to 24, 36, and 48 h. Axons extending to the lower surface of the insert were identified by immunostaining for β3-tubulin (MAB1195; R&D Systems). Neurite outgrowth was analyzed using Neuromath (Rishal et al., 2013).

### Antibodies and immunostaining

Primary antibodies used for Western blottings were as follows: rat anti-HA (Roche catalog 11-867-423), mouse anti-RPS5 (Abcam, ab58345), rabbit anti-RPS18 (Abcam, ab91293), rabbit anti-RPS15 (Abcam, ab90902), rabbit anti-RPL11 (Abcam, ab79352), rabbit anti-TUJ1 (Covance catalog MRB_435_P), mouse anti-Rpl22 (Santa Cruz Biotechnology, sc-373993), rabbit anti-gERK (Sigma, M5670), and rabbit anti-albumin (Cedarlane, CLAG5140).

Primary antibodies used for immunostaining were as follows: rat anti-HA (Roche catalog 11-867-423), mouse Y10B (Abcam catalog ab37144), rabbit anti-TUJ1 (Covance catalog MRB_435_P), rat anti-MBP (Millipore, MAB395-1ML), and chicken anti-NFH (Abcam, ab72996). Fluorescent secondary antibodies were from Jackson ImmunoResearch. Staining was observed using an Olympus FV1000 Confocal laser-scanning microscope at 40× or 60× magnification with oil-immersion Olympus UPLSAPO objective and was analyzed with FV10-ASW2.0 software.

### RiboTag analyses and RNA-seq

Cre driver lines were crossed with RiboTag mice to generate animals heterozygous for Cre and homozygous for the *Rpl22-HA* allele, except for *Advillin-Cre* crosses that were used as heterozygotes for both alleles. Tissues were extracted and HA immunoprecipitation (IP) was conducted as previously described (Sanz et al., 2009), with slight modifications. Briefly, DRG or neurons resuspended from culture were homogenized on ice in supplemented homogenization buffer [50 mM Tris (pH 7.0), 100 mM KCl, 12 mM MgCl_2_, 1% NP-40, and 1 mM DTT, 1.5× protease inhibitor cocktail, 300 units/ml RNasin (Promega), 150-µg/µl cyclohexamide, and 200 mM RVC] using 2-ml glass Teflon Potter Elv tissue grinders. After homogenization, lysates were transferred to Eppendorf tubes and centrifuged at 4°C for 10 min at 6,932 × g. Supernatants were collected to low bind tubes and 10% of each sample was set aside as input; 5 µg of anti-HA antibody was added to each sample and incubated on rotator for 4 h at 4°C. Magnetics beads were washed with the homogenization buffer and added to the Ab-homogenates mix, 100 µl of beads per sample, for overnight incubation at 4°C on rotor. Beads were washed three times, 5 min each, with high salt buffer [50 mM Tris (pH 7.0), 300 mM KCl, 12 mM MgCl_2_, 1% NP-40, and 1 mM DTT, 1× protease inhibitor cocktail, 200 units/ml RNasin, and 150-µg/µl cycloheximide]. After washes, RNA was eluted and purified using the Qiagen RNeasy micro kit including on-column DNase digestion.

RNA-seq libraries were prepared in three to five replicates per condition, using at least 5 ng of starting total RNA for each replicate. Processing was with Ribo-Zero Gold (Epicentre), using the Ovation RNA-seq system V2 (NuGEN). After library preparation, amplified double-stranded cDNA was fragmented into 125 bp (Covaris-S2) DNA fragments, which were (200 ng) end-repaired to generate blunt ends with 5’-phosphates and 3’-hydroxyls and adapters ligated. The purified cDNA library products were evaluated using the Agilent Bioanalyzer and diluted to 10 nM for cluster generation *in situ* on the HiSeq paired-end flow cell using the CBot automated cluster generation system. All samples were multiplexed into a single pool to avoid batch effects (Auer and Doerge, 2010) and sequenced using an Illumina HiSeq 2500 Sequencer (Illumina) across multiple lanes of 50-bp paired-end sequencing, corresponding to three samples per lane and yielding between 44 and 85 million reads per sample. Quality control was performed on base qualities and nucleotide composition of sequences, with removal of outliers. Alignment to the *Mus musculus* (mm10) refSeq (refFlat) reference gene annotation was performed using the STAR spliced read aligner (Dobin et al., 2013) with default parameters. On average, 83.7 ± 8% of the reads mapped uniquely to the mouse genome. Total counts of read-fragments aligned to candidate gene regions were derived using the HTSeq program (http://htseq.readthedocs.io/) with mouse mm10 (December 2011) refSeq (refFlat table) as a reference and used as a basis for the quantification of gene expression. Only uniquely mapped reads were used for subsequent analyses. Differential expression analysis was conducted with R-project and the Bioconductor package edgeR (Robinson et al., 2010). Statistical significance of the differential expression was determined at false discovery rate (FDR) <0.1 (Table 1).

RNA-seq data were deposited within the Gene Expression Omnibus (GEO) repository (www.ncbi.nlm.nih.gov/geo), accession numbers GSE102316 (RiboTag data from DRG ganglia) and GSE110374 (RiboTag data from neuronal cultures). The Matlab function “clustergram” was used for generating hierarchical trees of differentially expressed genes (DEGs) and generating heatmaps. The “cosine” option was used for distance measurement (“Pdist value”) using average linkage (“LinkageValue”). No standardization was used. Gene ontology (GO) term enrichment analyses were done with the Ontologizer GO analysis tool (Bauer et al., 2008; http://ontologizer.de/webstart/), using total genome entries as background and the topology-weighted algorithm to identify-enriched GO terms.

### Mass spectrometry analysis

Peak lists were generated using PAVA_Jul2009. Database search was performed by Protein Prospector v5.4.2 on UniProtKB.2009.12.15.random.concatdatabase, considering rat, mouse and human proteins only (344290/21095996 entries searched). Only tryptic peptides were considered, one miscleavage was permitted. Carbamidomethylation of Cys residues was considered as a constant modification in the analysis, while variable modifications included acetyl (protein N-term), acetyl + oxidation (protein N-term M), Gln->pyro-Glu (N-term Q), label: 13C(6)15N(2) (K), met-loss (protein N-term M), met-loss + acetyl (protein N-term M), and oxidation (M). Two variable modifications per peptide were permitted. Precursor mass tolerance was set at 15 ppm, and fragment mass tolerance was 0.8 Da. Acceptance criteria: minimal scores were 22 and 15, for proteins and peptides, respectively; max E values were 0.05 and 0.1, for proteins and peptides, respectively. Entry ID redundancy was addressed by translating the UniProt identifiers to UniRef90 (release date 2010-02-09, version 15.14), and a peptide list was generated accordingly. Sequences shared by multiple UniRef90 entries could not be used reliably for quantification, hence were removed from the list. The search engine was also used to calculate light/heavy peptide ratios. Mass survey “scans” were summed up considering a −10- to +30-s window around the time the precursor ion was selected for CID analysis. Peak areas representing the first three isotope peaks of the identified peptide and its counterpart serve as the basis for the calculation. The SNR threshold applied was 10, the mass resolution was matched to that set for the Orbitrap data acquisition, i.e., 15,000. Only the best ID was considered in each LC/MS analysis, yielding a single L/H value for each light/heavy pair even if both were identified from CID data.

Proteins that featured at least two unique peptides with quantitative information were used for further analysis. Ratios (H/L) were transformed to logarithmic scale with base 2 for calculation of median values. Percentages of incorporation were calculated from the medians.

## Results

### Cell type-specific ribosome pull-downs from DRG

**Figure 1.**
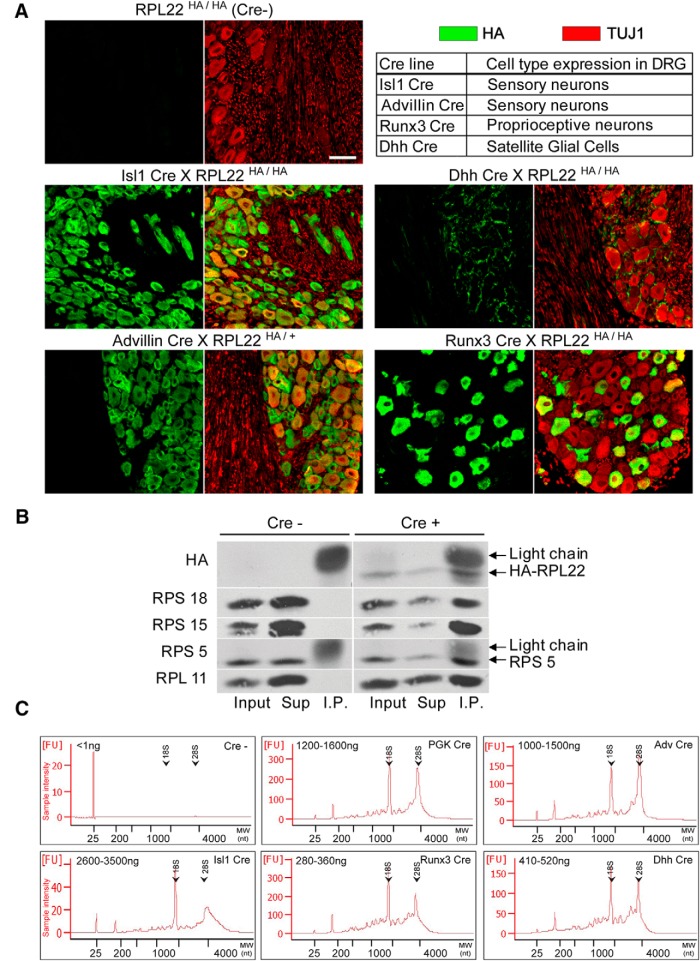
Cell type-specific ribosome isolation from DRG. ***A***, DRG sections from adult RiboTag mice with the indicated genotypes immunostained for HA (green, left) and the neuronal marker TUJ1 (red, right). Scale bar: 50 μm. ***B***, Isolation of sensory neuron ribosomal proteins from mouse DRG tissue. Total DRG isolated from four *Rpl22^HA/HA^* (Cre-) and four *Advillin-Cre X Rpl22^HA/+^* (Cre+) mice were extracted and IP with an HA antibody. Input, supernatant and IP fractions were then probed by Western blotting as indicated. Light chain indicates the precipitating HA antibody light chain. ***C***, Ribosomal RNA coprecipitation with Rpl22-HA from the indicated Cre RiboTag lines. Yields indicated in the upper left corner of each plot are from total DRG homogenate of one mouse (range for three repeats). The arrows indicate migration positions of 18S and 28S ribosomal RNA peaks. FU, fluorescence units.

Cell type-specific expression of Rpl22-HA was confirmed by HA immunostaining on DRG sections (Fig. 1*A*). To verify ribosome pull-down from neuronal tissues, HA pull-downs were analyzed for Rpl22-HA and for coprecipitation of additional ribosomal proteins, Rps18, Rps15, Rps5, and Rpl11 (Fig. 1*B*). Coprecipitating signal for Rpl22 and the four additional ribosomal proteins was detected in RiboTag ganglion pull-downs but not in wild-type controls, indicating that ribosomes are specifically pulled down only from Rpl22-HA expressing cells. We further examined RNA coprecipitation in RiboTag pull-downs from DRG extracts of all the Cre-RiboTag lines of interest. Both 18S and 28S ribosomal RNAs were found in all RiboTag pull-downs, compared to undetectable levels in pull-downs from Cre-animals (Fig. 1*C*). Hence, tagged Rpl22 is specifically expressed in the expected cell types in different Cre driver lines for peripheral ganglia, and Rpl22-HA pull-downs from extracts of whole ganglia can be used to analyze ribosome-associated RNAs in cell types of interest.

### Neuronal versus glial translatomes from DRG

**Figure 2.**
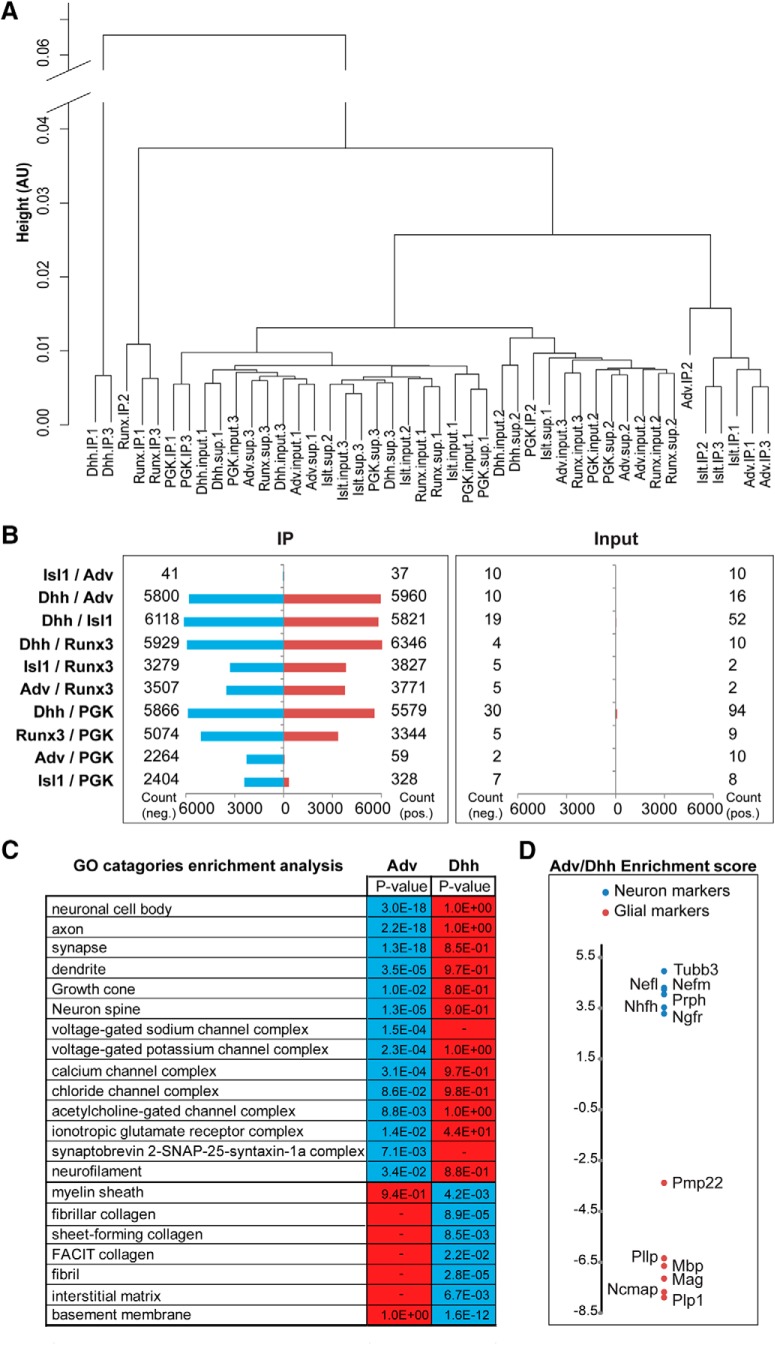
DRG cell type-specific pull-down of ribosome-associated RNAs. ***A***, Hierarchical clustering analysis shows that the mouse RiboTag IP samples cluster according to driver Cre identity, whereas input and supernatant samples do not. AU, arbitrary units. ***B***, Differential expression analysis of RNA-seq datasets from HA IPs from different Cre RiboTag lines. The differences between the input samples (right) are negligible, whereas marked differences are found for the IP datasets (left), except for the Isl1/Adv comparison. Red bars: upregulated transcripts; blue bars: downregulated transcripts. ***C***, GO enrichment analysis on IP datasets reveals clear distinctions between *Adv* versus *Dhh-Cre RiboTag mice*; *p* values for GO category enrichment are shown for each genotype. Blue shading indicates *p* < 0.05, red *p* > 0.05. ***D***, Enrichment analysis for specific neuronal (blue) versus glial (red) markers in Adv versus Dhh RiboTag datasets.

RNA-seq was then conducted on the IP, input and supernatant fractions described in Figure 2. Hierarchical clustering of the sequencing data distinguished IP from input samples, except for PGK-Cre, which is ubiquitously expressed in all tissues. Subclustering of the IP sample datasets followed the predicted cell type specificity of the different Cre drivers (Fig. 2*A*). Differential expression analysis between IP sample datasets further emphasized the unique composition of ribosome-associated ensembles derived from different Cre lines, except for the Isl1/Adv ensembles which are practically identical (Fig. 2*B*), due to the expression of these two Cre drivers in all sensory neuron subtypes in the DRG. Parallel comparisons of the input samples showed that they are mostly identical (Fig. 2*B*). Cell type specificity of the IPs was further confirmed by GO analysis on Adv-RiboTag (neuronal) versus Dhh-RiboTag (glial) datasets (Fig. 2*C*), and by comparison of enrichments of specific neuronal versus glial markers in both datasets (Fig. 2*D*). Thus, RiboTag IPs from DRG extracts enable characterization of RNA ensembles associated with ribosomes in neuronal versus glial cells.

**Figure 3.**
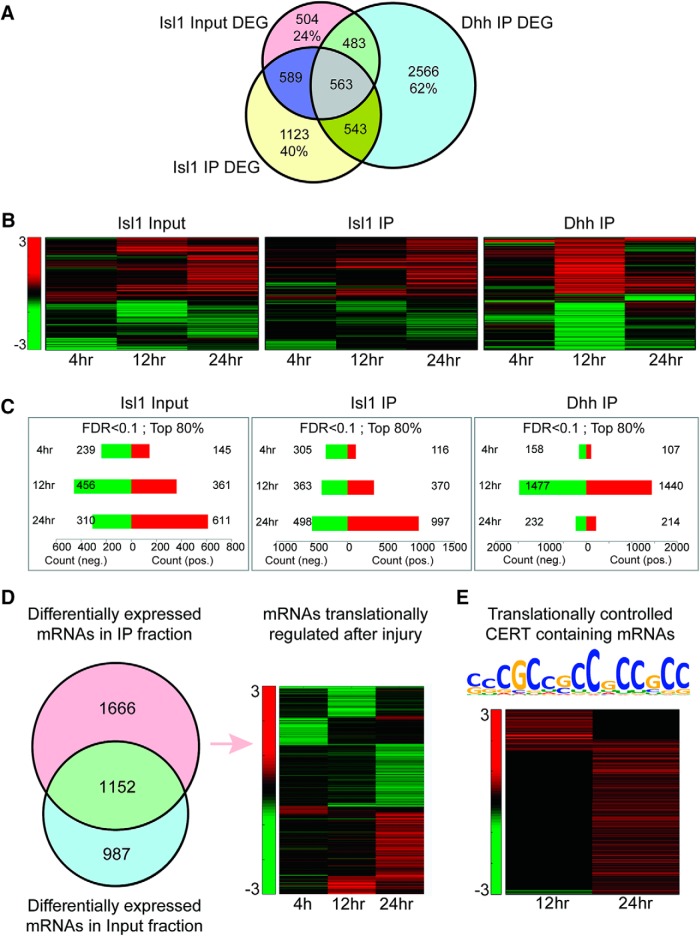
Translatome regulation in the DRG following SN injury. *Isl1-RiboTag* and *Dhh-RiboTag* mice underwent a sciatic lesion and L4-5-6 DRG were dissected at 4, 12, or 24 h after injury. HA-IP reaction was carried on the homogenate followed by RNA-seq analysis on the IP and input samples. Transcripts with FPKM higher than 1 were subjected to a differential expression analysis and compared to the naive samples at each time point. Only transcripts at an FDR lower than 0.1 were considered as differentially expressed. Differential expression in the input samples represents changes in total transcripts’ abundance in the DRG tissue. Differential expression in the IP samples represents changes in the association of transcripts with neuronal or glial ribosomes. ***A***, Comparison of differentially expressed genes (DEG) between the Input and Isl1 versus Dhh-RiboTag IP fractions. Percentages are the fraction of unique genes from the relevant dataset. ***B***, Clustering of the DEG shows different response dynamics over time in the different samples. ***C***, The number of down- and upregulated genes in each time point after filtering out the 20% least changing genes. ***D***, DEG lists from the IP and input fraction of the Isl1-Cre RiboTag IP were compared for common and unique entries. In pink are DEG only in the IP fraction that are considered as candidates for translational regulation. In blue are the DEG only in the input fraction. In green are DEG in both the IP and input. The heatmap depicts the translationally regulated genes at each time point. ***E***, Revised CERT motif for the neuronal DRG dataset shown above a heatmap depicting log fold change values for CERT-containing transcripts in the dataset. See also Extended Data Figures 3-1, 3-2.

We then proceeded to examine changes in neuronal versus glial translatomes in the DRG following SN injury, using lumbar DRG from Isl1-Cre versus Dhh-Cre RiboTag mice at 4, 12, and 24 h after injury. Data were filtered such that only transcripts with an fragments per kilobase of transcript per million mapped reads (FPKM) above 1 were considered for further analysis. A differential expression analysis was performed between each time point and naive control with an FDR cutoff of 0.1. These analyses revealed unique populations of DEGs in input, neuronal RiboTag IP and glial RiboTag IP ensembles (Fig. 3*A*; Extended Data Figure 3-1). 40% of the DEG in the neuronal translatome are unique to this ensemble, 62% of the DEG in the glia are unique, and 24% of the DEG in the input are not found to be regulated in the RiboTag IP fractions of either neurons or glia. The responses also clearly differ in their kinetics, with marked differences in the time course of DEG regulation in neuronal versus glial translatomes after injury (Fig. 3*B*,*C*). Finally, a specific comparison of input versus Isl1-Cre RiboTag fractions highlights over 1600 DEG that change only in the ribosome-bound ensemble, but not in the input (Fig. 3*D*). This large group of genes is most likely under translational regulation in the first 24 h after injury, with little or no change in their transcription at the time points examined.

10.1523/ENEURO.0276-17.2018.f3-1 Extended Data Figure 3-1 DEGs from RNA-seq of Input and RiboTag pull-downs of lumbar DRG after SN injury. Isl1-Ribotag, neuron-specific Cre, Dhh-Ribotag, glial Cre. Each spreadsheet lists the DEG for a different fraction-input fraction, Isl1 Ribotag pull-down, and Dhh Ribotag pull-down. Data include transcripts IDs, log fold change (logFC), *p* values, FDR values, FPKM values, counts, and normalized counts. Download Figure 3-1, XLSX file.

### Enrichment of the Eif4e-responsive cytosine-enriched regulator of translation (CERT) motif in the neuronal injury response translatome

We then applied MEME motif analysis software (Bailey et al., 2009) to search for motifs enriched in the translatome datasets. An initial screen revealed a number of short cytosine-enriched motifs, suggesting that a CERT motif might be overrepresented in the dataset. The CERT motif provides dose-dependent responsiveness to the translation initiation factor Eif4e (Truitt et al., 2015). We retrieved 5’UTR sequences for the previously defined CERT-containing genes (Truitt et al., 2015), and used these as a training set in MEME, revealing a shared motif highly similar to the published CERT (Fig. 3*E*). The revised motif is not completely identical to that previously published due to recent updating of a number of the 5’UTR sequences in the refSeq database.

To search for the CERT motif in our data, we retrieved the longest known refSeq 5’UTRs for all translationally regulated genes in four kinetic clusters. We then searched this set for CERT enrichment versus a background set of the 5’UTR sequences of all neuronal transcripts found in RiboTag pull-downs at FPKM > 1. The CERT motif was found to be enriched in translatome 5’UTRs at 12 and 24 h after injury and was present in 339 translationally regulated genes (Fig. 3*E*; Extended Data Figure 3-2). This analysis suggests that Eif4e may be involved in translational regulation of neuronal injury responses via the CERT motif.

10.1523/ENEURO.0276-17.2018.f3-2 Extended Data Figure 3-2 Transcripts that are translationally upregulated 12 and 24 h after injury and contain the CERT motif in their 5'UTR. The table lists transcript IDs, log fold change (LogFC), and FDR values for each time point after injury. Download Figure 3-2, XLSX file.

### Axon outgrowth proteomes reveal robust injury-regulated de novo protein synthesis

**Figure 4.**
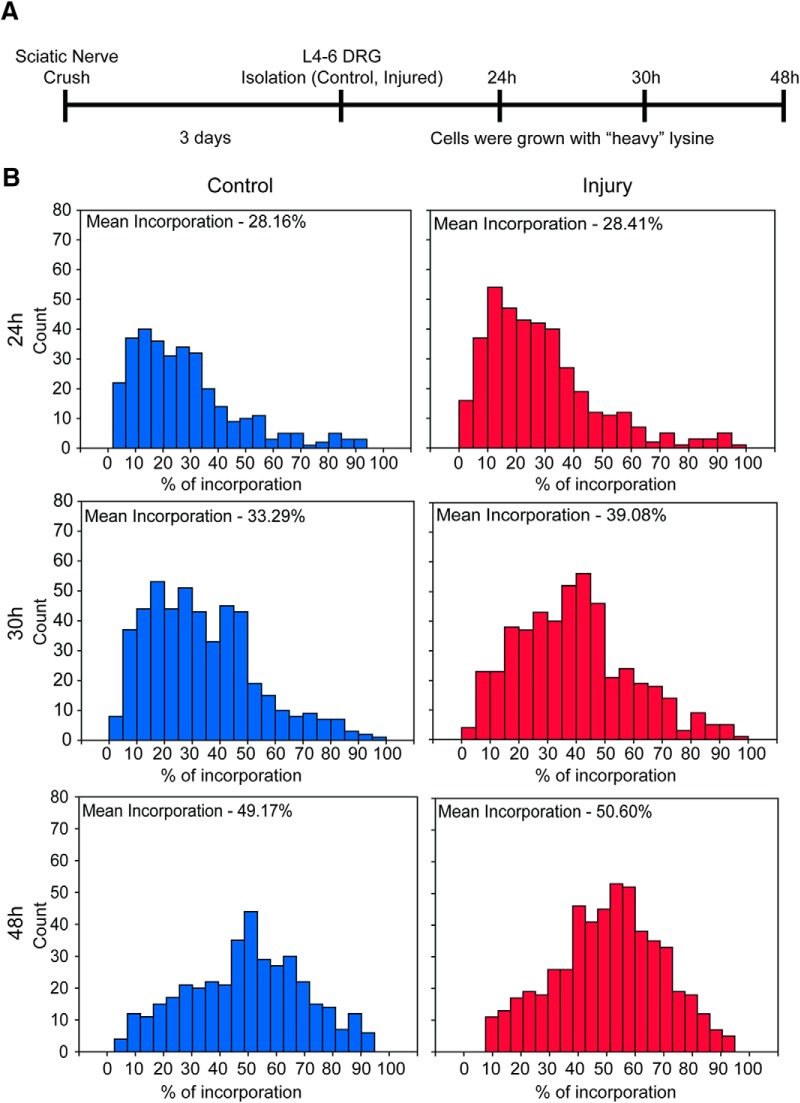
Incorporation of heavy lysine during neurite outgrowth. ***A***, Schematic outline of experimental paradigm for SILAC analyses in rat neurons. ***B***, Distribution of heavy lysine incorporation in control or conditionally injured neurons 24, 30, and 48 h after plating (neurite-enriched proteome). There was no difference in protein synthesis rates between control and conditionally-injured neurons.

**Figure 5.**
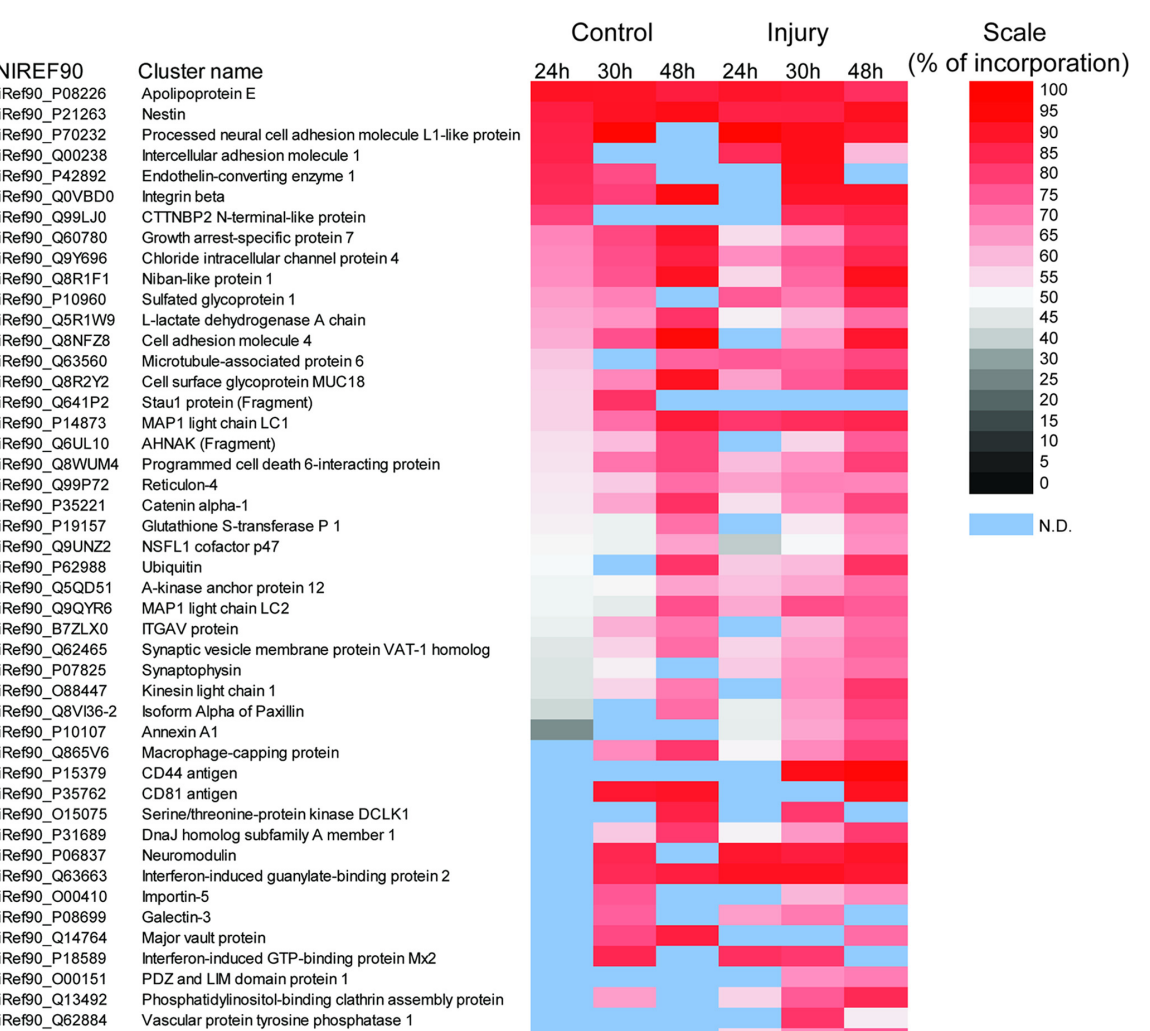
*De novo* synthesized proteins in sensory neuron outgrowth. Highly synthesized proteins identified in the early time points of rat DRG neuron outgrowth. A similar battery of highly synthesized proteins was found in control and in conditionally injured neurons. Each time point represents a combined sample of 75–100 animal equivalents. See also Extended Data Figure 5-1.

The findings detailed above suggest that translational regulation plays a role in the neuronal response to nerve injury. Conditional injury of SN is used as a model to differentiate between arborizing and elongating regenerative growth of DRG neurons in culture. Conditionally injured neurons initiate axon extension earlier in culture and have longer axons compared to the arborizing neurons at 24–48 h after plating. To characterize proteome differences between arborizing and elongating growth, we examined incorporation rates of heavy lysine (Lys8) into newly synthesized proteins in axons of compartmentalized rat DRG neuron cultures 24–48 h after plating (Fig. 4*A*). The average incorporation rate into axonal proteins did not differ significantly between conditionally injured versus control neurons over the time points examined, reaching ∼50% at 48 h after plating (Fig. 4*B*). However, we unexpectedly observed a subset of proteins with much higher incorporation rates, delineating an ensemble of axonal proteins robustly synthesized *de novo* during initial neurite extension (Fig. *5*; Extended Data Figure 5-1).

10.1523/ENEURO.0276-17.2018.f5-1 Extended Data Figure 5-1 Proteins with different translation rates in control and conditionally injured neurons. Proteins featuring at least two unique peptides with “heavy” lysine incorporation obtained from neurite enriched proteomes at 24, 30, and 48 h in culture. Download Figure 5-1, XLSX file.

### Transcription versus translation in axon outgrowth regulation

**Figure 6.**
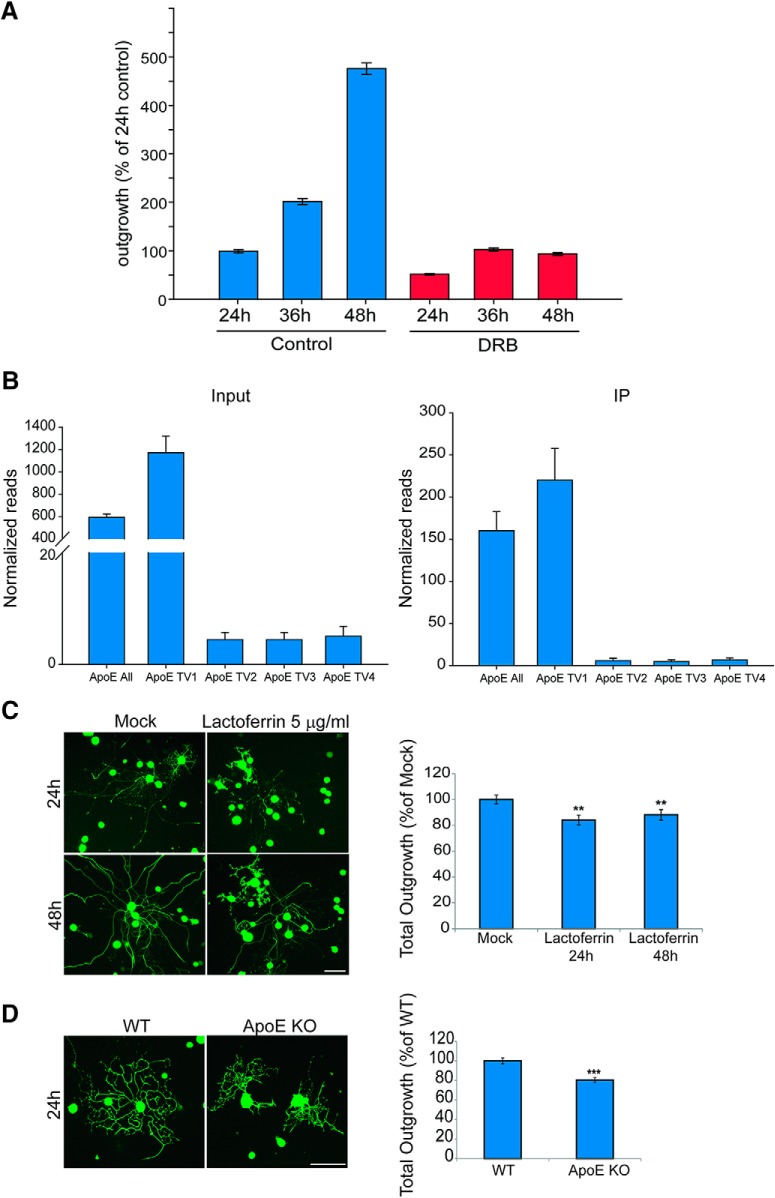
Transcription inhibition and ApoE effects on neurite outgrowth. ***A***, Neurite outgrowth comparison for mouse DRG neurons 24, 36, and 48 h after plating with or without transcription inhibitor (DRB) for first 12 h. All groups were normalized to 24 h control (*n* = 3, Mean ± SEM). ***B***, Expression levels of different ApoE transcript variants (TV) quantified by Nanostring analyses of input and HA-RiboTag pull-downs from wild-type and Adv-Cre-RiboTag DRG. The variants analyzed were ApoE All (GenBank refSeq NM_001305844.1), ApoE TV1 (NM_009696.4), ApoE TV2 (NM_001305819.1), which contains a CERT-like motif, ApoE TV3 (NM_001305843.1), and ApoE TV4 (NM_001305844.1). Data analysis was conducted in nSolver3.0, with normalization to Tubb3 as a reference gene. A large proportion of the most highly expressed ApoE variants are associated with ribosomes, supporting ApoE translation during axonal growth. ***C***, YFP-expressing adult mouse DRG neurons were treated with 5-μg/ml lactoferrin and then allowed to grow for 48 h. Representative images at two time points are shown on the left; scale bar: 100 μm. Quantification of total neurite outgrowth from three independent experiments reveals a significant decrease in axon growth after lactoferrin treatment (right panel). Mean ± SEM, *n* ≥ 300 neurons per experimental group; **p < 0.01 (ANOVA with Holm–Sidak method). ***D***, DRG neurons from adult wild-type or ApoE KO male mice grown in culture for 24 h and then fixed and stained for NFH (green). Representative images from ApoE KO and WT neurons are shown on the left; scale bar: 100 μm. Quantification of total neurite outgrowth from four independent experiments reveals a significant decrease in axon growth in ApoE KO DRG neurons. Mean ± SEM, *n* ≥ 250 neurons per experimental group; ***p < 0.001 (Student’s *t* test).

We further examined the contribution of transcription versus translation in driving axonal outgrowth by culturing DRG neurons in Boyden chambers in the presence of the transcription inhibitor DRB for the first 12 h in culture. DRB was then removed and axon outgrowth was quantified at 24, 36, and 48 h, revealing that initial axonal outgrowth can proceed despite reduced transcription (Fig. 6*A*). This suggests that the robustly translated mRNA subset identified above may be recruited from previously transcribed transcripts that are translated on need. ApoE, a protein best known for its link to Alzheimer’s disease (Yu et al., 2014), is one of the most robustly synthesized candidates at the initial stage of axon growth, and one of the ApoE 5’UTR splice variants harbors a CERT-like motif. Interestingly, a previous study described ApoE protein upregulation in SN injury, but did not determine the cell type involved or the role of the upregulated ApoE in the nerve (Melemedjian et al., 2013). Others have reported that exogenous application of ApoE or ApoE modulators can have a beneficial effect on neurite outgrowth (Kosacka et al., 2009). We verified the endogenous synthesis of ApoE in mouse DRG sensory neurons by RiboTag pull-downs from neuronal cultures and quantification of ribosome-associated ApoE RNA splice variants by Nanostring digital expression analysis (Kulkarni, 2011). As shown in Figure 6*B*, a single ApoE variant is predominant in DRG neurons, and is robustly associated with ribosomes in the pull-downs. Notably, the predominant Tv1 splice variant is not the one that harbors a CERT-like motif (Tv2; Fig. 6*B*). To assess whether the neuronal synthesis of ApoE might affect neurite outgrowth, we then tested the effects of the ApoE receptor inhibitor lactoferrin on DRG neuron outgrowth in serum-free cultures. Lactoferrin indeed had a modest inhibitory effect on neurite outgrowth, which was more pronounced at the earlier time point (Fig. 6*C*). Moreover, DRG neurons from ApoE null mice showed a modest yet significant decrease in axon outgrowth in culture (Fig. 6*D*). These data support the possibility that endogenously synthesized ApoE is secreted by adult sensory neurons for autocrine enhancement of their own axon growth.

**Table 1.** Statistics

Data	Data structure	Type of test	Power	Notes
Figure 2B	GO enrichment analysis	Ontologizer 2 (topology-weighted algorithm)	*p* < 0.05	Standard settings as described in Bauer et al. (2008)
Figure 3C	Contrast analysis	EdgeR Genewise Negative Binomial Generalized Linear Models	FDR < 0.1	Genes with FPKM < 1 were filtered
Figure 3E	RNA motif occurrence	MEME (FIMO tool) uses dynamic programming algorithm to convert log-odds scores into *p* values, assuming zero-order background model	*p* < 0.0001	Standard settings as described in Bailey et al. (2009); Grant et al. (2011)
Figure 6C	Normally distributed	ANOVA with Holm–Sidak	*p* < 0. 01	
Figure 6D	Normally distributed	Student’s *t* test	*p* < 0.001	
Extended Data Figures 3-1, 7-1	Differential expression analysis	EdgeR Genewise Negative Binomial Generalized Linear Models	Quantile-adjusted conditional maximum likelihood (qCML)	TMM normalization, genes with FPKM < 1 were filtered

**Figure 7.**
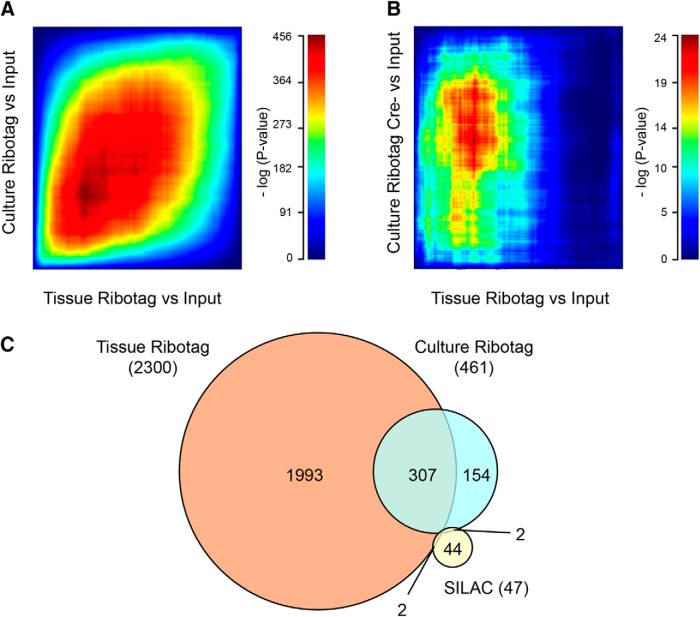
Comparison of datasets. Rank-rank hypergeometric overlap (RRHO) heatmaps comparing RiboTag-enriched genes from (***A***) cultured DRG neurons and L4-6 DRG tissue (*Runx3-Cre* driver used for both) and (***B***) Cre- cultured DRG neurons and *Runx3-Cre+* L4-6 DRG tissue. Compared gene lists were sorted by directional *p* values and plotted as -log10 transformed hypergeometric test *p* values. Overlap across *Runx3-Cre+* samples (cultured neurons and tissue) was significant (***A***), whereas poorer consistency was observed when correlating *Runx3-Cre* tissue to Cre- DRG neurons (***B***). ***C***, Overlap of genes significantly enriched by Runx3 RiboTag in DRG tissue, Runx3 RiboTag in DRG culture (Extended Data Figure 7-1) and axonal proteins with high translation rates identified by SILAC (Fig. 5). Total number of significantly enriched genes in respective datasets is shown in parentheses. Twofold enrichment in IP versus input and FDR < 0.1 were set as cutoffs to define RiboTag-enriched genes.

We further analyzed concordance of the different datasets in this study by generating a RiboTag dataset from proprioceptive sensory neurons cultured for 24 h, using Runx3-Cre RiboTag cultures. As shown in Figure 7, there is significant overlap in the ribosome-associated RNA ensemble in culture versus *in vivo* (Fig. 7*A–C*; Extended Data Figure 7-1), while there is minimal overlap of both RiboTag datasets with the small SILAC dataset of highly synthesized axonal proteins (Fig. 7*C*).

10.1523/ENEURO.0276-17.2018.f7-1 Extended Data Figure 7-1 Genes significantly enriched in RiboTag pull-downs from proprioceptive neurons extracted from ganglia and cultures (RNA-seq). Lists of overlapping genes between Runx3-Cre RiboTag ganglia, Runx3-Cre RiboTag culture RNA-seq, and SILAC datasets. Download Figure 7-1, XLSX file.

## Discussion

The above findings reveal multifaceted layers of translational regulation in the neuronal response to injury and subsequent regenerative outgrowth. Translatome-wide analyses in models of neuronal injury or regeneration have mainly focused on compartmentalized translation within injured or regenerating processes (Baleriola and Hengst, 2015; Twiss et al., 2016; Rangaraju et al., 2017; Terenzio et al., 2018). Our RiboTag data reveal widespread translational regulation in the somatic response to injury, most prominently in a subset of upregulated ribosome-associated transcripts containing the CERT motif for Eif4e. This suggests that increased Eif4e activity may fuel the injury response in peripheral neurons, and Eif4e can indeed be rate limiting in mRNA translation (Truitt et al., 2015). Moreover, regulation of Eif4e or its binding proteins has been reported in a number of neuronal trauma or pain models (Chen et al., 2007; Ayuso et al., 2013; Khoutorsky and Price, 2018). Eif4e activity can be regulated by phosphorylation, interaction with different partners or upregulation, offering diverse opportunities to target the pathway (Moy et al., 2017). However, direct targeting of Eif4e for enhancing neuronal regeneration would be a high-risk strategy, since increasing Eif4e levels or activity promotes cellular transformation and cancer cell survival (Truitt et al., 2015; Hu et al., 2016).

In addition to highlighting regulatory aspects of translation in neuronal regeneration, our SILAC analyses identified a subset of transcripts that are robustly translated in the initial stages of axonal outgrowth. Although our SILAC data cannot discriminate between greatly increased synthesis versus very rapid turnover, a recent study that systematically analyzed protein turnover rates in different cell types did not report high turnover in neurons (Mathieson et al., 2018). Early studies suggested that the first phase of axon outgrowth is transcription-independent in sensory neurons (Smith and Skene, 1997), and our findings support that observation, while showing that later stages of outgrowth require new transcription. The data are consistent with initial translation of a cohort of preexisting mRNAs in the early injury response, perhaps ensuring a rapid reaction to lesion before transcriptional changes. The minimal overlap between our axonal SILAC and ganglia or whole-neuron RiboTag datasets might suggest that the SILAC experiment highlighted proteins synthesized within axons. The data may also reflect the inherent technical limitations of using a basic SILAC approach in non-dividing cells, since it was difficult to identify a time point allowing sufficient labeling that also revealed clear differences between conditionally lesioned and naive cultures. Future work in such models should use methods that do not require extensive labeling or pre-incubation periods, such as recently described methods based on O-propargyl-puromycin (OPP) labeling (Barrett et al., 2016; Forester et al., 2018), that can also be conducted in tissues and potentially also *in vivo* (Terenzio et al., 2018).

ApoE is one of the *de novo* synthesized proteins identified in our analyses. This protein is most prominently studied for its roles in Alzheimer’s disease (Yu et al., 2014) but is also implicated in a number of studies of peripheral neuronal injury and regeneration (Kosacka et al., 2009; Li et al., 2010; Melemedjian et al., 2013). Our data suggest that ApoE may have autocrine effects on axon growth, whereby growth-promoting ApoE is synthesized and secreted by the neuron to facilitate its own axon outgrowth. Future studies on this translationally regulated cohort of mRNAs are likely to provide additional new insights on the mechanisms underlying growth and regeneration of injured neurons.

## Synthesis

Reviewing Editor: Robert Kalb, Northwestern University Decisions are customarily a result of the Reviewing Editor and the peer reviewers coming together and discussing their recommendations until a consensus is reached. When revisions are invited, a fact-based synthesis statement explaining their decision and outlining what is needed to prepare a revision will be listed below. The following reviewer(s) agreed to reveal their identity: Andrew Chisholm

Both reviewers found the work well done, albeit mostly descriptive in nature. It is a starting point for further studies on the translational control of axonal regeneration. Reviewer #2 was particularly concerned about the lack of follow up investigations, for example regarding the CERT motif. IN addition this reviewer felt that the two data sets are not well integrated except in the most vaguest of ways. Although experiments investigating the CERT motif would be useful, at the very least the authors need to better integrate the ribotag and SILAC data. Its too rudimentary as it stands. IN addition, directly addressing the editorial and disucssion points (raised by both reviewers) is needed .

Specific comments by reviewer #1

eNeuro review (eN-NWR-0276-17) “Translational Regulation in Initial Stages of the Neuronal Injury Response”

Summary

This manuscript addresses translational regulation in the early neuronal injury response using translatome and proteomic analyses in rodent dorsal root ganglia and sensory neurons. The authors identified >1600 genes that are translationally regulated after nerve injury, potentially involving the translation initiation factor Eif4e based on the observation many of these gene products contain 5’UTR CERT motif. Further, the authors state ApoE is robustly synthesized de novo after nerve injury, suggesting ApoE may be a key injury response factor.

The translatome analyses constitute a large data set of interest to the axon regeneration community. The work appears in large part technically careful and thorough. However, the presentation is in places wuite confusing and could be improved (see comments). The authors devote considerable attention to ApoE, but it is unclear if ApoE is itself regulated by injury.

Major comments

1. As seen in Figure 5, ApoE (and many other proteins) is abundant in control neurons and after injury. While ApoE may indeed be relevant for axon regrowth, it is not clear that its synthesis is regulated by injury.

2. The data as presented do not support “prominent roles for translational regulation in the neuronal injury response”, although enrichment of the 5’UTR CERT motif suggests translational regulation. If this cannot be shown, the authors should tone down the abstract and title. For example, the functional importance of the CERT motif has not been tested. Does the ApoE transcript have CERT motif?

3. The authors state in methods that they used Ribotag mice for translatomic analyses and Wistar rats for proteomic analyses. It would be good to explicitly state the model organisms used for each results and figure. The authors could expand on the rationale for switching between mice and rats for these experiments.

4. The authors analyzed the differential expression of samples from Adv vs Dhh in Figure 2 (cell-specific pull-down), but compared Isl1 vs Dhh in Figure 3 (translatome regulation following injury). Do the authors assume both Adv and Isl1-expressing cells the same sensory neurons, ie have identical profiles? Some rationale for the different comparisons would help.

Minor comments

1. Figures 1-4. Axis labels and units are frequently missing or uninformative.

2. Figure 1C: What do the arrowheads represent? What is [FU]?

3. Figure 2: Dhh.IP.2 is missing from the hierarchical clustering analysis. What are the units for ‘height’? (y-axis)

4. Figure 2: Figure 2D is not referred to in the text. Is that important?

5. Figure 3: What do the three columns in each sample mean? Are they coming from different time point?

6. Figure 3: Do the samples in 3B and 3C match? Please provide label for each square (ie. “Isl1 input” for the first square? etc.)

7. Figure 5: What does “scale (%)” represent? Percent upregulation or downregulation? If so, relative to what?

8. Figure 6: clarify the nomenclature ApoE TV1, TV2 etc. Do these correspond to the ApoE subtypes ε1- ε4?

9. Figure 6: Use one-way ANOVA with post-test, instead of t-test, in Fig 6C.

10. There are numerous typos throughout the manuscript.

Confidential Review Collaborator

Reviewer #2

This study explores translational regulation following axonal injury, using RiboTag in the DRG in vivo and SILAC in sensory axons in vitro. The design, validation, and execution of the RiboTag experiments are sound and have generated data sets of potential interest. Enrichment of CERT motifs is suggested as evidence of potential regulation by Eif4e, while the robust production of ApoE (detected by SILAC) is suggested to support axon growth.

The strength of this study is the experimental execution of the two profiling methods, resulting in reliable data sets that generate hypotheses for further study, with the RiboTag the stronger of the two. The manuscript in current form is quite limited in its generation and testing of such hypotheses.

The biggest drawbacks of the study fall into 4 categories:

(1) The presence of the CERT motif raises an interesting hypothesis regarding control by Eif4e that is not further experimentally assessed in any system.

(2) The two techniques are utilized in systems different enough from one another as to offer only mild advantage of being presented together, and no cross-platform analyses or insights are suggested beyond the general theme that they both relate to translational control in neurons after injury. The distinct ways in which they may complement each other are described only in vague terms.

(3) The lack of differential control of axonal proteins between control and conditioning lesion is surprising and suggest that finding a useful time point in this experimental setup may be challenging. The authors’ alternate analysis of “highly synthesized proteins” in both conditions is hard to interpret. While some likely play a role in growth and/or reflect unregulated translation in response to injury, might many others simply be proteins that have high turnover?

(4) The in vitro analysis of ApoE receptor blockade adds only modestly to studies implicating ApoE in axon growth and lacks further validation.
